# Frequency of* TNFA*,* INFG*, and* IL10* Gene Polymorphisms and Their Association with Malaria* Vivax* and Genomic Ancestry

**DOI:** 10.1155/2016/5168363

**Published:** 2016-11-24

**Authors:** Adriana Antônia da Cruz Furini, Gustavo Capatti Cassiano, Marcela Petrolini Capobianco, Sidney Emanuel Batista dos Santos, Ricardo Luiz Dantas Machado

**Affiliations:** ^1^Department of Dermatologic, Infectious, and Parasitic Diseases, College of Medicine of São José do Rio Preto, São José do Rio Preto, SP, Brazil; ^2^University Center of Rio Preto, UNIRP, São José do Rio Preto, SP, Brazil; ^3^Laboratory of Tropical Diseases–Prof. Luiz Jacintho da Silva, Department of Genetics, Evolution and Bioagents, University of Campinas, Campinas, SP, Brazil; ^4^Department of Biology, São Paulo State University, São José do Rio Preto, SP, Brazil; ^5^Laboratory of Human and Medical Genetics, Federal University of Pará, Belém, PA, Brazil; ^6^Laboratory of Basic Research in Malaria, Section of Parasitology, Evandro Chagas Institute, Belém, PA, Brazil

## Abstract

Polymorphisms in cytokine genes can alter the production of these proteins and consequently affect the immune response. The trihybrid heterogeneity of the Brazilian population is characterized as a condition for the use of ancestry informative markers. The objective of this study was to evaluate the frequency of -*1031T>C*,* -308G>A and -238G>A TNFA*,* +874 A>T IFNG* and -*819C>T, and -592C>A IL10* gene polymorphisms and their association with malaria* vivax* and genomic ancestry. Samples from 90* vivax* malaria-infected individuals and 51 noninfected individuals from northern Brazil were evaluated. Genotyping was carried out by using ASO-PCR or PCR/RFLP. The genomic ancestry of the individuals was classified using 48 insertion/deletion polymorphism biallelic markers. There were no differences in the proportions of African, European, and Native American ancestry between men and women. No significant association was observed for the allele and genotype frequencies of the 6 SNPs between malaria-infected and noninfected individuals. However, there was a trend toward decreasing the frequency of individuals carrying the* TNF-308A* allele with the increasing proportion of European ancestry. No ethnic-specific SNPs were identified, and there was no allelic or genotype association with susceptibility or resistance to* vivax* malaria. Understanding the genomic mechanisms by which ancestry influences this association is critical and requires further study.

## 1. Introduction

With the completion of the Human Genome Project and the ease of identifying variations in DNA using currently available tools, several studies on genetic associations have evaluated the genetic bases of certain traits (e.g., the susceptibility to clinical manifestations of various types of diseases, including diabetes, cancer, and hypertension, as well as autoimmune, infectious parasitic, and cardiac diseases) [1–6]. Thus, based on the genetic variability, these association studies are based on comparisons of the allele and/or genotype frequencies of some SNPs present in candidate genes between a group who have the disease or the outcome of interest and an unaffected group [7, 8].

Malaria is one of the most studied infectious diseases. It is the primary parasitic disease worldwide and is responsible for approximately 214 million cases annually, resulting in more than 438,000 deaths [9]. Currently, it is widely accepted that genetic factors of the human host contribute to the infection and different clinical manifestations of the disease [10–12]. The observed genetic variants associated with malaria include those present in erythrocytes, which play an essential role as host cells during the asexual life cycle of the parasite [13–15]. Moreover, polymorphisms in cytokine genes can alter the production or activity of these proteins and consequently affect the inflammatory response to malaria [16–18]. As a result, these polymorphisms may be associated with susceptibility to or progression of disease [17, 19].

The prognosis of* Plasmodium* infection depends on the balance between pro- and anti-inflammatory cytokines [20–23]. IFN-*γ*, TNF-*α*, IL-6, IL-12, IL-1*β*, and IL-8 are reported at higher levels in individuals infected with* Plasmodium* than in controls or in individuals with severe malaria [21, 24, 25]. However, contradictory results have also been observed, with lower levels of these cytokines reported in infected patients [25, 26].

TNF-*α* participates in tumorigenesis, apoptosis, immune cell activation, hyperthermia [18, 22], and parasitemia reduction [27, 28]. However, it can play different, concentration-dependent roles in malaria, ranging from protection against the destructive activity of infection on the vascular and brain endothelium to changes in blood glucose levels [29, 30]. SNPs in this gene have the potential to alter transcription factors, influencing the circulating levels of the cytokine [16]. The A (-308) and C (-1031) alleles have been associated with circulating levels of the cytokine and with clinical symptoms but not with susceptibility [27], whereas the G allele (-308) has been associated with increased susceptibility to malaria* vivax* [19]. Other alleles at positions -1031T, -863C, -857T, -308G, and -238G have been associated with an increased risk of developing cerebral malaria in patients in Myanmar [31].

IFN-*γ* acts as a regulator of antigen presentation, proliferation, and differentiation in lymphocyte populations and plays a modulatory role in the immune response mediated by anti-inflammatory cytokines [32], such as IL-10. In malaria, this cytokine is believed to play roles in both pathogenesis and protection [33]. Some studies have evaluated potential associations between* IFNG* gene polymorphisms (including SNP +874A>T) and malaria and found no association with susceptibility to* vivax* malaria [17] or severity of* P. falciparum* infections [34]. A recent study also found no association between the presence of SNP +874A>T and the antibody response against* P. vivax* blood-stage proteins [35]. However, in Brazil, individuals infected with* P. vivax* carrying the AA genotype showed lower levels of IFN-*γ* [21].

IL-10 cytokine has a negative immunoregulatory effect [36, 37] on IL-1, IL-6, IL-8, IL-12, IFN-*γ*, and TNF-*α* [17, 27] that is essential for maintaining homeostasis and limiting tissue damage by infectious agents [34]. The production of the IgG, IgA, and IgM isotypes induced by IL-4 is synergistic with IL-10 [38]. However, high levels can contribute to the maintenance of the parasite in the host and can be related to cerebral malaria and high levels of parasitemia [20, 21, 24]. The proportion of C alleles of SNPs -819 and -592 was lower in individuals infected with* P. vivax* than in healthy individuals. Furthermore, individuals carrying the genotypes -819CC and -592CC had lower levels of circulating IL-10 [17]. By contrast, another study conducted in the Brazilian Amazon found no association between the presence of these SNPs and susceptibility to* P. vivax* [21].

Thus, certain aspects of these observed associations have proven irreproducible in subsequent studies performed in different populations [39–41], with contradictory results for different SNP associations with susceptibility to different* Plasmodium* species and levels of circulating cytokines and antibodies. There are many reasons for the lack of consistency in these results, but discrepancies are often due to population stratification, which can occur in populations with different allele frequencies between and within subgroups [8]. If the population subgroups are represented in different proportions between individuals of the case and control groups, then spurious associations may be observed; thus, ancestry informative markers (AIMs) have been employed in an attempt to avoid the population stratification problem [42, 43].

This consideration is particularly important in studies involving admixed populations, as is the case in the Brazilian population due to crosses involving primarily Europeans, Africans, and Native Americans. Previous studies employing AIMs in Brazil demonstrated that the allele distributions in genes involved in pharmacokinetics [44, 45] or in the costimulation of B and T lymphocytes [46] were affected by the proportions of genetic ancestry. The frequencies of several cytokine gene alleles vary significantly among some ethnic groups and geographic populations. Moreover, the lack of data on Native Americans in the Brazilian population motivated us to investigate the frequency of polymorphisms in* TNFA*,* INFG,* and* IL10* genes in people living in a malaria-endemic area of the Brazilian Amazon and their possible association with malaria* vivax* and genomic ancestry.

## 2. Materials and Methods

### 2.1. Sample

The sample used in this study was from the municipality of Goianésia do Pará, Pará state (03°50′33′′S; 49°05′49′′W), Brazil, which is a malaria-endemic area in the Brazilian Amazon. The sample was a subset of the individuals analyzed in Cassiano et al. [46]. A total of 141 unrelated individuals older than 14 years were recruited at the Goianésia do Pará Malaria Diagnosis Center. Of these individuals, 90 were diagnosed with* vivax* malaria by microscopy, and infection was subsequently confirmed using nested-PCR; no infections by any human malaria species were observed in the remaining 51 individuals. All participants or guardians signed the consent form, and the project was approved by the Goianésia do Pará health authorities and by the Research Ethics Committee (CAAE 01774812.2.0000.5415) of the College of Medicine of São José do Rio Preto (Faculdade de Medicina de São José do Rio Preto).

### 2.2. DNA Extraction and Genotyping

DNA was extracted using an Easy-DNA™ extraction/purification kit (Invitrogen, CA, USA), according to the manufacturer's specifications.

#### 2.2.1. *TNFA* Genotyping

Three different SNPs [-238G>A (rs361525), -308G>A (rs1800629), and -1031T>C (rs1799964)] were genotyped within the promoter region of* TNFA* gene through PCR-RFLP method according to the following conditions and primers. For the -308G>A position (rs1800629), were used the oligonucleotides forward 5′-GAG GCA ATA GGT TTT GAG GGC CAT-3′ and reverse 5′-GGG ACA CAC AAG CAT CAAG-3′. A quantity of 100 ng of DNA was used with 1x buffer (20 mM Tris-HCl [pH 8.4], 500 mM KCl), 5% of glycerol, 1.5 mM MgCl_2_, 0.2 *μ*M of each dNTP, 0.6 pmol of each primer, and 0.5 U of* Taq* Platinum DNA Polymerase (Invitrogen, São Paulo, Brazil). The amplification process consisted of an initial denaturation step of 94°C for 5 min and 35 denaturing cycles (94°C for 30 s, 59°C for 30 s, and 72°C for 1 min), which was followed by a final extension at 72°C for 5 min. The PCR products were visualized on a 2% agarose gel stained with 2.5% GelRed™ (Biotium, Hayward, USA). The PCR products at 147 bp were digested with* Nco*I (Fermentas, Vilnius, Lithuania) restriction endonuclease for 3 hours at 37°C to identify the genotypes [47]. The digestion products were stained with 2.5% GelRed (Biotium, Hayward, USA) and viewed on a 12.5% polyacrylamide gel after ethidium bromide staining. The resulting fragment for the AA genotype was 147 bp, while the fragments for the GG genotypes were 126 and 21 bp, and those for the GA genotypes were 147, 126, and 21 bp [47].

The following oligonucleotides were used for the* TNFA*-1031T>C SNP (rs1799964): forward 5′-TAT GTG ATG GAC TCA CCA GGT-3′ and reverse 5′-CCT CTA CAT GGC CCT GTC TT-3′. Genomic DNA (100 ng) was amplified with 0.5 U of* Taq* Platinum DNA polymerase (Invitrogen, São Paulo, Brazil), 1.5 mM MgCl_2_, 0.2 *μ*M of each dNTP, and 0.6 pmol of each primer. Polymerase chain reactions were run for 35 cycles: 5 min at 94°C, 30 s at 57°C, and 1 min at 72°C, followed by a final extension at 72°C for 5 min. These oligonucleotides generated a 251 bp fragment visualized on a 2% agarose gel stained with 2.5% GelRed (Biotium, Hayward, USA). The product (10 *μ*L) was digested with 0.5 *μ*L of* Bbs*I (Fermentas, Vilnius, Lithuania) at 37°C for 12 h, subjected to electrophoresis in a 12.5% polyacrylamide gel after ethidium bromide staining, resulting in 251 and 13 bp fragments for the TT genotype; 251, 180, 71, and 13 bp fragments for the TC genotype; and 180, 71, and 13 bp fragments for the CC genotype [47].

The PCR and RFLP reactions for the* TNFA*-238G>A position (rs361525) were standardized according to the protocols of Hedayati et al. [48]. The following oligonucleotides were used: forward 5′-ATC TGG AGG AAG CGG TAG TG-3′ and reverse 5′-AGA AGA CCC CCC TCG GAA CC-3′. Briefly, amplification was performed in a final volume of 25 *μ*L containing 100 ng of DNA, 0.5 U of* Taq* Platinum Polymerase (Invitrogen, São Paulo, Brazil), 1.5 mM MgCl_2_, 0.2 *μ*M of each dNTP, and 0.6 pmol of each primer. The amplification reactions were performed under the following conditions: initial denaturation for 5 min at 94°C; 35 cycles of 30 s at 94°C, 30 s at 60°C, and 1 min at 72°C; and a final extension of 5 min at 72°C, which generated a 153 bp fragment that was visualized on a 2% agarose gel stained with 2.5% GelRed (Biotium, Hayward, USA). A total of 10 *μ*L of the PCR product was subjected to restriction enzyme digestion with MspI (Thermo Scientific) using 0.5 *μ*L of the required enzyme at 37°C for 15 min. The genotypes were identified as AA for the 156 bp fragment, GG for the 133 bp fragment, and GA for 153 and 133 bp fragments in a 2% agarose gel stained with 2.5% GelRed (Biotium, Hayward, USA).

#### 2.2.2. *IL10* Genotyping

For the* IL10* SNPs at the -592C>A (rs1800872) and -819C>T positions (rs1800871), the reactions were standardized in-house with the following oligonucleotides: forward 5′-GGG TGA GGA AAC CAA ATT CEC-3′ and reverse 5′-GAG GGG GTG GGC TAA ATA TC-3′. The 25 *μ*L PCR mixture contained 100 ng of DNA, 0.5 U of* Taq* Platinum Polymerase (Invitrogen, São Paulo, Brazil), 1.5 mM MgCl_2_, 0.2 *μ*M of each dNTP, 0.6 pmol of each primer, and 5% glycerol. The cycling conditions were as follows: 94°C for 5 min; 35 cycles of 94°C for 30 s, 54°C for 30 s, and 72°C for 1 min; and a final extension at 72°C for 10 min. These oligonucleotides generated a 361 bp fragment. For the* IL10*-819C>T SNP, the PCR products were digested overnight at 37°C with 0.5 *μ*L of* RseI* (Fermentas, Vilnius, Lithuania). For the* IL10*-592C>A SNP, 10 *μ*L of the PCR product was digested with 0.5 *μ*L of the enzyme* RsaI* (Invitrogen, CA, EUA). After digestion, the fragments generated at the -592C>A position were 240, 77, 36, and 8 bp for the AA genotype; 317, 36, and 8 bp for the CC genotype; and 317, 240, 77, 36, and 8 bp for the CA genotype. At the -819 position, the TT, CC, and TC genotypes were identified with 270 and 91 bp; 217, 91, and 53 bp; and 270, 217, 91, and 53 bp bands, respectively. The 2% agarose gel stained with 2.5% GelRed (Biotium, Hayward, USA).

#### 2.2.3. *IFNG* Genotyping

The polymorphism at the +874A>T position in the* IFNG* gene (rs2430561) was identified using allele-specific oligonucleotide-polymerase chain reaction (ASO-PCR) according to Flori et al. [30], with modifications. The oligonucleotides used were:* IFNG* (+874) CP: 5′-TCA ACA CTG ATA AAG CTC AC-3′,* IFNG* (*+874*)* T*: 5′-TTC TTA CAA CAC AAA ATCAAA TCT-3′, or* IFNG* (*+874*)* A*: 5′-TTC TTA CAA CAC AAA ATC AAA ATC-3′. These oligonucleotides resulted in a 264 bp fragment after changing the annealing conditions from 56°C for 40 s to 53°C for 1 min by Flori et al. [30].

The amplified product was analyzed using electrophoresis on a 2% agarose gel stained with 2.5% GelRed (Biotium, Hayward, USA). The AA genotype was identified when a 264 bp fragment was observed in the electrophoresis of the A allele tube, and the TT genotype was identified with the presence of a 264 bp fragment for the T allele tube. For the AT genotype, one 264 bp fragment was observed in each of the two reaction tubes (A and T).

### 2.3. Determination of Ancestry

Individual ancestry estimates were based on a panel of 48 insertion-deletion (InDel) ancestry informative markers (AIMs) as described in Santos et al. [49]. The ancestry data for the samples from Goianésia do Pará were previously presented in a larger subset of samples in Cassiano et al. [46]. The AIMs were genotyped in three multiplex reactions with 16 markers in each reaction, and electrophoresis was performed on a capillary sequencer (ABI®3130 Genetic Analyzer, Applied Biosystems) under the conditions described by de Seixas Santos Nastri et al. [50]. A standard ladder (ABIGS LIZ-500, Applied Biosystems) was used in each sample as a reference for the identification of InDel markers. All of the investigated AIMs significantly differed in frequency in populations of different geographical origins.

The individual proportions of European, African, and Native American ancestry were estimated in the program Structure v2.3.4 using the Admixture Model with a 100,000 burn length and 100,000 iterations after burning; the allele frequencies were independently modeled [51]. For the ancestry estimates, the data obtained in the investigated sample were plotted against the parental population data that formed the Brazilian population, which included Amerindian (246), Western European (290), and Sub-Saharan African (201) individuals.

### 2.4. Statistical Analysis

All statistical analyses were performed using R software. The allele, genotype, and haplotype frequencies and deviations from the Hardy-Weinberg equilibrium were estimated using the SNPassoc package [52]. Differences in the ancestry proportions between genotypes were determined using fitted logistic regression models for age, gender, and infection status. A similar analysis was performed to evaluate differences in ancestry proportions among the different haplotypes using the haplo.glm function [53]. Binary logistic regression was used to graphically explore the associations between the polymorphisms and ancestry proportions using the multinom package [54]. Differences in the genotype and haplotype frequencies between the infected and noninfected individuals were tested using the SNPassoc package with adjustment for the covariates age, gender, and ancestry. In all multivariate analyses, the SNPs were included following different genetic models (codominant, recessive, dominant, and additive). *p* values < 0.05 were considered significant.

## 3. Results

### 3.1. Epidemiological Characteristics of the Study Participants

The demographic data of the subjects included in the study are listed in Table 1. Of the 141 participants, 90 (63.8%) had mild malaria, and 51 (36.2%) individuals were not infected at the time of collection. The proportion of men was higher in the group with malaria (74.4%) than in group of noninfected individuals (56.9%) (*p* = 0.03). Additionally, the proportion of individuals that reported previous episodes of clinical malaria was higher in the group of malaria-infected individuals (91.1% versus 68.6%, *p* < 0.01). Age, number of previous malaria episodes, and proportion of genetic ancestry (European, African, and Native American) were similar between the two groups. The ancestry data were previously presented in a larger cohort by Cassiano et al. [46], who showed that the main contribution was European (44.2%), followed by African (31.8%) and Amerindian (24.0%) contributions. There were no differences in the proportions of African, European, and Native American ancestry by gender (*p* = 0.99, 0.65, and 0.48, respectively, Mann-Whitney *U*-test).

### 3.2. Genotype and Haplotype Distributions

The genotype and allele distributions of the studied SNPs are shown in Table 2. The* IFNG*+874A>T SNP was successfully genotyped in 92.2% of the samples. When the allele and genotype frequencies of the six SNPs were compared between malaria-infected and noninfected individuals, no significant association was observed. All SNPs were at Hardy-Weinberg equilibrium in both groups (all *p* values > 0.05) (Table 2). We conducted the tests following the additive, dominant, recessive, and heterozygous models, and the lowest *p* values are shown in Supplementary Table 1 (in Supplementary Material available online at http://dx.doi.org/10.1155/2016/5168363). Although the highest* AA* genotype frequency was observed for the* IFNG+*874A>T SNP in the group of malaria-infected individuals, this difference did not reach the significance level (OR = 1.87, 95% CI: 0.91–3.82, *p* = 0.08).

Haplotype analyses were performed for the three SNPs in the* TNFA* gene and for the two SNPs in the* IL10* gene. Four haplotypes in the* TNFA* gene were responsible for more than 98% of all potential combinations. The* TNFA*-1031T>C SNP was in moderate linkage disequilibrium with the* TNFA*-308G>A and -238G>A SNPs (*D*′ = 0.70 and 0.67, resp.), whereas the* TNFA*-308G>A and -238G>A SNPs exhibited a *D*′ of 0.85. For the* IL10* gene, strong linkage disequilibrium occurred between the -819C>T and -592C>A SNPs and three haplotypes were observed. The comparison of the haplotype frequencies between the malaria-infected and noninfected individuals is shown in Table 3.

### 3.3. Association between Polymorphisms and Genetic Ancestry

**Figure 1:**
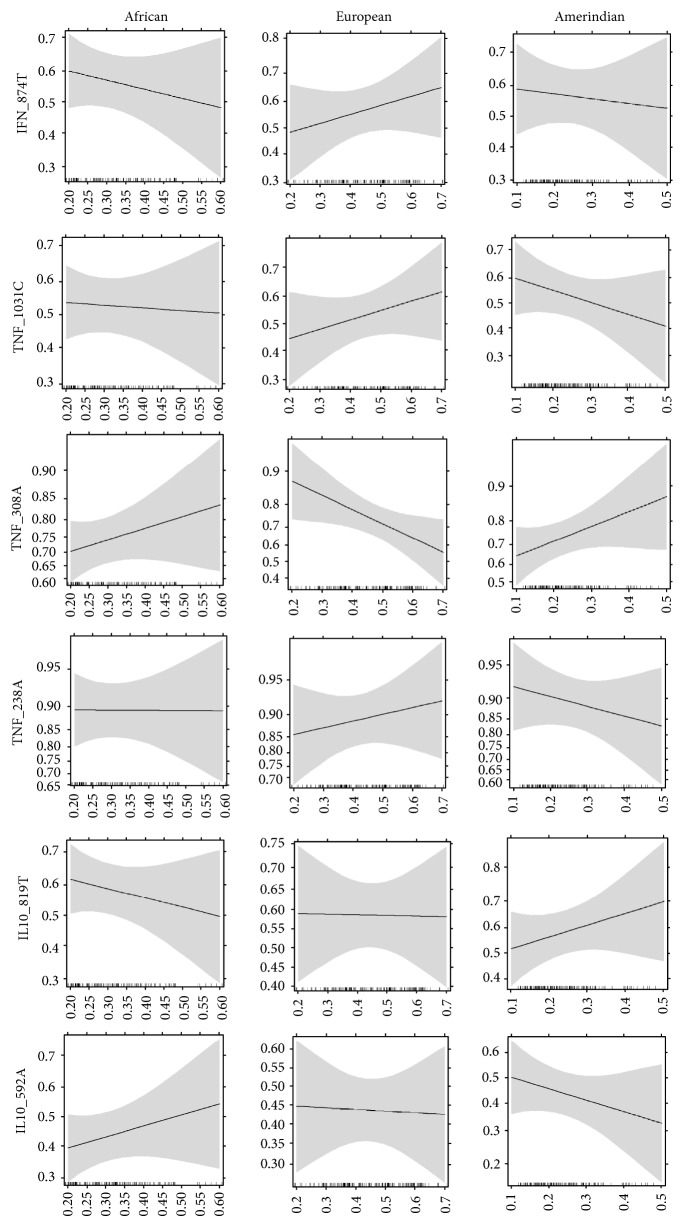
Binary logistic regression model used to evaluate the frequency of individuals carrying the mutant allele of all analyzed SNPs relative to the individual proportions of genetic ancestry. The shading around the lines represents the 95% confidence interval. The graph was constructed using the ggplot2 package in the R program.

The individual proportions of the African, European, and Native American genetic ancestries were analyzed as continuous variables. In the present study, no differences were observed in the mean proportion of any ancestry among the different genotypes and haplotypes analyzed (Table 4 and Supplementary Table 2).  Figure 1 shows the graphical representation of the binary logistic regression model used to evaluate the frequency of individuals carrying the mutant allele of all analyzed SNPs in relation to the individual genetic ancestry proportions. The frequency of individuals carrying the* TNFA-308A* allele progressively decreased with the increasing proportion of European ancestry (*p* = 0.03). However, when the Bonferroni correction for multiple tests was used, this association was no longer significant (*p* = 0.18). No other association was observed.

## 4. Discussion

Previous studies reported different allele frequencies in cytokine genes among different ethnicities [18, 55–57]. Due to these studies and the participation of these proteins in numerous processes related to the pathogenesis of various diseases, we evaluated the frequencies of polymorphisms in the* TNFA*,* IFNG*, and* IL10* genes in a highly admixed Brazilian population and related their distributions to the proportions of genetic ancestry using AIMs. We selected a population from northern Brazil where there was a higher contribution of Native American ancestry due to the lack of data in studies of this nature involving indigenous populations [58]. Because these cytokines play a key role in the modulation of the immune response in malaria, we evaluated whether these polymorphisms were related to protection against* vivax* malaria. However, this study did not provide evidence of such associations.

The -*308G>A* SNP (rs1800629) is located in the promoter region of the gene, and the presence of the *A* allele forms a binding site for the AP1 transcription factor that has been associated with increases in TNF-*α* production [18]. The frequency distribution of the *A* allele observed in our study (13.83%) was similar to that observed in previous studies in the Brazilian population (12–16%) [59–61]. According to data from the 1000 Genomes project, the frequency of the *A* allele is similar between Europeans (13%) and Africans (12%). This finding was in agreement with our results because no differences were observed in the frequencies of this allele according to the proportions of genetic ancestry. Contradictory results were observed for malaria, with the* TNFA-308A* allele associated with higher susceptibility/severity [62–64], without alterations [39] or with resistance to* P. falciparum* malaria [65]. Regarding* vivax* malaria, which was the focus of the present study, our results were in agreement with other studies, including those in the Brazilian Amazon that did not observe any associations between the* TNFA-308G>A* SNP and susceptibility or clinical manifestations due to* P. vivax* infection [10, 19, 66].

The -*238G>A* SNP (rs361525) does not have a clearly established function but seems to affect the circulating cytokine levels because it is located on a repressor site in the* TNFA* gene [16]. The 5.38% frequency of the A allele (-238) in our results was similar to the data for Europeans and Africans, which ranged from 4 to 6% [67]. The frequency of the presence of the A allele at the -238 and -376 positions is low worldwide. In the Brazilian Amazon, previous indices ranged from 5 to 7% [19, 68], and no associations were described with* vivax* malaria in Pará [19]. In contrast, the GA genotype was associated with psoriasis in southeastern Brazil [69], and the A allele was associated with a decrease in* falciparum* malaria parasitemia in Burkina Faso [30], cerebral malaria in Kenya [70], and malarial anemia [62]. This SNP was associated with increased susceptibility to vivax malaria in the Amazon region only when evaluated in the TATGG haplotype (-1031/-863/-857/-308/-238) [19].

The 24.82% frequency of the C allele at the -1031 position (rs1799964) of the* TNFA* gene is similar to data from the 1000 Genomes project (15% and 21% for Africans and Europeans, resp.) and in other Brazilian population (27.9%) [68]. In malaria, this SNP was associated with cytokine levels and clinical symptoms but not with susceptibility in India [27]. The C allele is associated with a twofold higher chance of cerebral malaria caused by* P. falciparum* [71] in Thailand. In Africa, the CC genotype is associated with repeated malaria episodes by* P. falciparum* [47, 65] and the T allele is associated with high parasitemia [30]. In Brazil, the CC genotype is associated with protection against leprosy but not malaria* vivax* [68].

One hypothesis for the lack of association of the evaluated SNPs is that malaria can occur due to possible linkage disequilibrium of the SNPs in* TNFA* with the human leukocyte antigens (HLAs), which can cause nonfunctional mutations [65, 66]. The A allele (-308) is described as having a strong linkage disequilibrium with HLA-Bw53 and DRB1^*∗*^1302-DQB1^*∗*^0501, whereas the A allele at the -238 position of the* TNFA* gene appears to be linked to HLA-B53 but with different immune characteristics [62]. The haplotype frequencies in cytokine genes can vary extensively among different ethnic groups most likely due to selective pressure on the human genome and thus affect the susceptibility and clinical outcomes of diseases such as malaria [36]. This effect might have affected our results due to the admixture observed in the Brazilian population.

The gene sequence of this cytokine is highly conserved, with few polymorphisms. The SNP at the -183G>T position is related to increased transcription activity [26], whereas +874A>T is located in a region where the number of replicates can modulate the expression of messenger RNA and the production of cytokines [21, 72]. The T allele is associated with a high number of replicate copies and activates the transcription site for the NF-*κ*B pathway, which correlates with high cytokine expression [73, 74]. The AA, TA, and TT genotypes are associated with low, intermediate, and high production of IFN-*γ*, respectively [21, 75].

The highest frequency of the A allele (*IFNG*+874) is described in individuals with European ancestry and is 46% (http://hapmap.ncbi.nlm.nih.gov/). Indeed, the evaluated population in the present study had a European contribution of almost 50% [46], and the frequency of this allele was detected in 67.3% of the evaluated sample. However, no association was detected with any ancestry or with malaria. Studies conducted in the United States with African-American and Caucasian populations found higher frequencies of 66% and 37% [76] and 48% and 25% [77], respectively. Our data showing the higher frequency of the mutant A allele are in agreement with studies in the Brazilian Amazon that found frequencies of 70.13% [21] and 73% [17] but all lacked an association with malaria caused by* P. vivax* or* P. falciparum*. Few studies have described an association between this SNP with malaria; however, its association with dermatitis was observed in India [78]. Importantly, higher levels of this cytokine allow a better immune response against obligate intracellular pathogens; thus, low frequencies of A allele may be associated with susceptibility to the disease.

The* IL10* gene has more than 27 polymorphic sites associated with SNPs that result in the differential production and expression of the cytokine [17, 36, 79], autoimmune and inflammatory diseases [80], bacterial [81] and viral infections [75], and human malaria [21]. The allele distributions for T (-819) and A (-592) in our results were 35.4% and 31.2%, respectively; these distributions were higher in Europeans than in Africans but lacked significant associations. These data disagree with those from the 1000 Genomes project [67], which report a higher frequency of the mutant alleles in Africans. Lokossou et al. [82] reported higher frequencies (41.53% and 41.31% for the T and A alleles in SNPs -819 and -592, resp.) for* falciparum* malaria in Benin. The allele and genotype distributions of SNPs in* IL10* are described as variables according to ethnic group [21, 36] and the A (-592), T (-819), and A (-1082) alleles are more frequent among African-Americans [82, 83]. Another study by Moraes et al. [80] did not find significant differences in the frequencies of genotypes, alleles, and haplotypes of five* IL10* SNPs (–3575, –2849, 2763, –1082, and –819) between the Brazilian and Dutch populations. However, studies of indigenous populations are scarce. In Brazil, a study of indigenous people from the Terena tribe of the state of Mato Grosso do Sul showed that the frequency of mutant alleles (-819T and -592A) was significantly higher than in individuals residing in Rio de Janeiro [84]. By contrast, we obtained the lowest rates for this ancestry, and no association was observed.

The CC genotypes for the two SNPS were associated with a decrease in IL-10 levels and low parasitemia in northern Brazil [17], which agreed with our data indicating no significant association with susceptibility to malaria. Two studies in Pará state, Brazil, also described no haplotype associations of the* IL10* gene with malaria [19, 21] and* falciparum* malaria in Africa [36]. In Piracicaba, southeastern Brazil, these SNPs were associated with chronic periodontitis in Caucasians [85]. Future analyses of parasitemia and cytokine indices may identify associations between the SNPs in the evaluated sample. One hypothesis for the lack of association is that the patients involved in the present study did not have malarial complications caused by* P. vivax*. Additionally, the transmission profile of the malaria of the area investigated could have had an effect, and the epidemiology was different from that observed in Africa. Another explanation may be the low frequency of some genotypes in the present study. Thus, the sample size may have been too small to find any possible association. This finding warrants further investigation.

## 5. Conclusion

The evaluation of ancestry informative markers (AIMs) allows estimations of admixtures at the individual level and avoids possible confounding factors due to ethnicity, such as in the trihybrid population sample evaluated in this study. The polymorphisms in the* TNFA*,* IFNG*, and* IL10* genes investigated in this study did not significantly differ according to ancestry and were not associated with risk or protection against* vivax* malaria. However, there was a decreasing trend in the frequency of the A allele with increasing proportion of European ancestry. In Brazil, this is the first study to evaluate the distribution of these genes according to ancestry. The results support the application of ancestry informative markers in future studies.

## Supplementary Material

Supplementary Table 1 describes tests of the genetic association of the polymorphisms between vivax malaria-infected and non-infected individuals.
In table 2 the genotype frequencies and proportions of African, European and Native American ancestry according to genotype are reported.

## Figures and Tables

**Table 1:** Characteristics of the study population.

Characteristic	Mild *vivax* malaria (*n* = 90)	Noninfected (*n* = 51)	*p* value
Gender, male^a^	74.4	56.9	0.03
Age (years)^b^	32.5 (23.75–43.5)	37.0 (26.0–45.0)	0.62
Genetic ancestry^c^			
European	0.442 ± 0.130	0.449 ± 0.130	0.76
African	0.318 ± 0.120	0.295 ± 0.112	0.26
Native American	0.240 ± 0.094	0.256 ± 0.111	0.35
Previous malaria episodes^b^	5.0 (2.0–7.0)	2.0 (0–6.0)	0.06
Previous history of malaria^a^	91.1	68.6	<0.01

^a^Percentage.

^b^Median (IQR).

^c^Mean ± SD.

**Table 2:** Distribution of the genotypes between *vivax* malaria-infected and noninfected individuals.

Gene	SNP	Genotype	Malaria			Noninfected			*p *value
			*n* (%)	MAF	HWE	*n* (%)	MAF	HWE	
*IFNG*	*+874A>T*	*AA*	39 (48.7)	0.30	0.91	17 (34.0)	0.37	0.08	0.22
		*AT*	34 (42.5)			29 (58.0)			
		*TT*	7 (8.8)			4 (8.0)			

*TNFA*	*-1031T>C*	*TT*	51 (56.7)	0.28	0.11	24 (47.1)	0.28	0.14	0.53
		*TC*	37 (41.1)			25 (49.0)			
		*CC*	2 (2.2)			2 (3.9)			

*TNFA*	*-308G>A*	*GG*	69 (76.7)	0.12	0.21	35 (68.7)	0.18	0.69	0.14
		*GA*	21 (23.3)			14 (27.4)			
		*AA*	0			2 (3.9)			

*TNFA*	*-238G>A*	*GG*	80 (88.9)	0.06	0.22	46 (90.2)	0.05	0.71	0.70
		*GA*	9 (10.0)			5 (9.8)			
		*AA*	1 (1.1)			0			

*IL10*	*-819C>T*	*CC*	39 (43.3)	0.34	0.87	19 (37.2)	0.35	0.15	0.55
		*CT*	41 (45.6)			28 (54.9)			
		*TT*	10 (11.1)			4 (7.8)			

*IL10*	*-592C>A*	*CC*	41 (45.6)	0.29	0.05	20 (39.2)	0.34	0.21	0.62
		*CA*	45 (50.0)			27 (52.9)			
		*AA*	4 (4.4)			4 (7.8)			

MAF: minor allele frequency; HWE: Hardy-Weinberg equilibrium.

*p* values were calculated from a chi-squared test.

**Table 3:** Haplotype frequencies in the *TNF* and *IL10* genes in *vivax* malaria-infected and noninfected individuals.

Haplotype	Malaria infected	Noninfected	OR (95% CI)	*p* value
*TNFA*_−1031/−308/−238_				
T/G/G	0.632	0.555	Reference	0.11
C/G/G	0.195	0.220	0.63 (0.30–1.31)	0.45
T/A/G	0.113	0.161	0.48 (0.20–1.13)	0.17
C/G/A	0.037	0.049	0.62 (0.15–2.35)	0.75

*IL*10_−819/−592_				
C/C	0.642	0.647	Reference	0.94
T/A	0.291	0.343	0.80 (0.44–1.44)	0.33
T/C	0.054	0.009	7.19 (0.89–57.7)	0.06

Odds ratios (OR), 95% confidence interval (CI).

**Table 4:** Haplotype frequency and its association with the proportions of African, European, and Native American ancestry.

Haplotype	Frequency	African			European			Native American		
		Proportion	Difference (95% CI)	*p* value	Proportion	Difference (95% CI)	*p* value	Proportion	Difference (95% CI)	*p* value
*TNFA*_−1031/−308/−238_										
T/G/G	0.615	0.31	Reference		0.44	Reference		0.25	Reference	
C/G/G	0.191		−0.01 (−0.05–0.03)	0.61		−0.01 (−0.06–0.03)	0.57		0.01 (−0.02–0.05)	0.49
T/A/G	0.121		−0.05 (−0.10–0.00)	0.05		0.06 (0.00–0.11)	0.05		−0.02 (−0.06–0.02)	0.37
C/G/A	0.044		−0.01 (−0.08–0.06)	0.78		−0.02 (−0.11–0.06)	0.55		0.06 (−0.03–0.15)	0.17

*IL*10_−819/−592_										
C/C	0.650	0.30	Reference		0.45	Reference		0.25	Reference	
T/A	0.303		−0.01 (−0.04–0.03)	0.67		0.00 (−0.04–0.04)	0.94		0.01 (−0.02–0.04)	0.55
T/C	0.040		0.01 (−0.07–0.08)	0.86		−0.02 (−0.10–0.06)	0.60		0.02 (−0.05–0.08)	0.63

The effects of each haplotype were relative to the most frequent haplotype used as a reference. Δ% indicates relative change in the ancestry proportions compared to the reference haplotypes with 95% confidence intervals.
