# Supplementary material for: Frequency of TNFA, INFG, and IL10 Gene Polymorphisms and Their Association with Malaria Vivax and Genomic Ancestry
Source: Mediators Inflamm. 2016 Nov 24;2016:5168363. doi: 10.1155/2016/5168363 (PMC5143728; doi:10.1155/2016/5168363)
Supplement: Supplementary file 1 — Supplementary Table 1 describes tests of the genetic association of the polymorphisms between vivax malaria-infected and non-infected individuals. In table 2 the genotype frequencies and proportions of African, European and Native American ancestry according to genotype are reported. [file 5168363.f1.pdf]

Suppementary Files

Supplementary Table 1. Tests of the genetic association of the polymorphisms between vivax malaria-infected and non-infected individuals.

Gene	SNP	Model	Genotype	OR (95% CI)	*p*-value	*p*-value (adjusted)[a]
IFNG	*-874A>T*	Heterozygous	AT vs AA/TT	0.54 (0.26-1.09)	0.08	0.08
TNF	*-1031T>C*	Additive	TT vs TC vs CC	0.69 (0.37-1.29)	0.24	0.23
TNF	*-308G>A*	Additive	GG vs GA vs AA	0.60 (0.30-1.22)	0.13	0.14
TNF	*-238G>A*	Dominant	GG vs GA/AA	1.15 (0.37-3.57)	0.81	0.61
IL10	*-819C>T*	Heterozygous	CT vs CC/TT	0.69 (0.34-1.37)	0.28	0.36
IL10	*-592C>A*	Additive	CC vs CA vs AA	0.76 (0.42-1.35)	0.35	0.16

[a]Adjusted for gender, age and previous history of malaria.

Odds ratios (OR), 95% confidence interval (CI)

Supplementary Table 2. Genotypic frequencies and proportions of African, European and Native American ancestry according to genotype.

Genotype	Frequency (%)	African[a]	European[a]	Native American[a]
IFNG-874A>T	n=129			
A/A	43.4	0.321 ± 0.124	0.431 ± 0.137	0.247 ± 0.107
A/T	48.8	0.306 ± 0.119	0.448 ± 0.124	0.246 ± 0.100
T/T	7.8	0.297 ± 0.080	0.497 ± 0.132	0.206 ± 0.098
P		0.82	0.35	0.49
TNFA-1031T>C	n=140			
T/T	52.9	0.308 ± 0.116	0.456 ± 0.126	0.236 ± 0.091
T/C	44.3	0.310 ± 0.121	0.435 ± 0.138	0.254 ± 0.111
C/C	2.8	0.347 ± 0.096	0.376 ± 0.012	0.277 ± 0.091
P		0.74	0.36	0.54
TNFA-308G>A	n=140			
G/G	74.3	0.317 ± 0.114	0.430 ± 0.127	0.253 ± 0.101
G/A	24.3	0.292 ± 0.130	0.482 ± 0.134	0.226 ± 0.098
A/A	1.4	0.272	0.550	0.178
P		0.59	0.07	0.19
TNFA-238A>G	n=140			
A/A	89.3	0.310 ± 0.121	0.447 ± 0.128	0.243 ± 0.098
A/G	10.0	0.301 ± 0.091	0.423 ± 0.150	0.268 ± 0.124
G/G	0.7	0.337	0.411	0.252
P		0.98	0.78	0.68
IL10-819C>T	n=140			
C/C	41.4	0.320 ± 0.121	0.445 ± 0.136	0.235 ± 0.099
C/T	48.6	0.298 ± 0.108	0.447 ± 0.126	0.259 ± 0.100
T/T	10.0	0.328 ± 0.148	0.431 ± 0.132	0.241 ± 0.108
P		0.54	0.92	0.53
IL10-592C>A	n=140			
C/C	43.6	0.322 ± 0.120	0.443 ± 0.138	0.235 ± 0.097
C/A	50.1	0.297 ± 0.106	0.447 ± 0.122	0.257 ± 0.099
A/A	5.7	0.341 ± 0.182	0.433 ± 0.151	0.226 ± 0.136
P		0.32	0.92	0.42

[a]Ancestry expressed as the mean ± SD
